# Dentists Constitutionally Treating Patients

**Published:** 1881-07

**Authors:** 


### ARTICLE IV.

### *Dentists Constitutionally Treating Patients.*

An interesting and instructive discussion took place at the meeting of the Odontological Society, on April 4th, between Mr. Stocken and Mr. S. J. Hutchinson, on the advisability and propriety of dental surgeons undertaking the constitutional treatment of their patients. The former gentleman delivered an essay "On the Value of certain Remedies in the Constitutional Treatment of Inflammatory Conditions of the Vascular Tooth Structures, and of Neuralgia arising therefrom," in the course of which he remarked that he was of opinion that dental surgeons did not generally give sufficient attention to the constitutional treatment of the cases under thair care. Mr. S. J. Hutchinson very properly differed from this opinion, and maintained that it was a mistake for a dental surgeon to undertake constitutional treatment, and that if this appeared necessary he should communicate with the patient's ordinary medical attendant and leave the details of the treatment to him. Any other course, he thought, would inevitably lead to strained relations between dental and medical practitioners, especially in country places.

That dental licentiates, as soon as the legality of their license was firmly established, would begin by degrees to assume the functions of medical practitioners, no one of any experience ever for one moment doubted, and therefore the discussion referred to above will cause but little surprise to those who have taken any interest in dental legislation. We presume that these gentlemen referred to dental surgeons who possessed no medical or surgical qualification, and not to registered practitioners practising dentistry, and must express our admiration of the manly and straightforward manner in which Mr. Hutchinson denounced a system which we cannot help thinking would, if established, be fraught with danger and likely to lead to the greatest abuse. Most of us will agree with the President that it is difficult to define the exact border-line between medical and dental practice, but few will agree with him that so long as there is any prospect of saving a tooth, the dental surgeon is justified in using any means at his disposal, whether constitutional or local, with this object. The fact is, that dental surgery ought never to have been separated from the main branch, of which it should be an integral part, of equal importance with ophthalmic or aural surgery; and the President went far to admit this when he said that he thought every dental practitioner should have a thorough knowledge of the value of constitutional remedies in dental practice, and in order to promote this he would be glad to see a chair of pharmacology attached to every dental school. It is difficult to perceive any real difference between granting a dental license to practise dental surgery, and granting an obstetric, ophthalmic, or aural license to practise obstetric, ophthalmic, or aural surgery; and inasmuch as we should strongly condemn the establishment of any fresh special licenses, so we feel bound to condemn the already existing dental license, as the outcome of the most short-sighted and mischievous legislation that it has been our lot to witness. No fault is found with the dental license, any more than with the obstetric license or the diploma in state health, so long as they are only granted as additional qualifications; but we do strongly object to them being granted as licenses to practise; and surely the course of events has justified this objection on our part. If gentlemen possessing the license in dentistry only are at liberty to prescribe for the constitutional maladies of their patients, then there is no use whatever in taking the trouble to obtain a license in medicine. It was for this very reason that the obstetric license, which some time since was a registerable qualification, was deprived of its licensing power and rendered merely an additional qualification. Let us hope that the words of the President of the Odontological Society of Great Britain will bear fruit, and that every dental practitioner may in the future have a thorough knowledge of the value of constitutional remedies in dental practice. The only way to accomplish this end is to make the dental license of the future an additional qualification only. It is a remarkable thing that dental licentiates themselves do not insist upon this, instead of allowing themselves to be entirely separated from, and outside the pale of the profession of medicine, of which they *ought* to be a branch.

At present dentistry in this country is divided into two great sections—viz., those registered medical practitioners who elect to practise as dental specialists, and whose names are to be found on the British Medical Register; and those dental-licentiates, non-qualified dentists, herbalist-dentists, bone-setter-dentists, barber-pharmacist-dentists, etc., whose names are to be found mixed up in a promiscuous manner on the Dental Register. These two sections in reality have no connection whatever with each other, the one being a medical specialty, followed by registered medical practitioners mostly possessing the additional special qualification of Licentiate in Dental Surgery; the other being a calling to itself, outside the pale of the profession, and followed by gentlemen who hold no registerable medical qualification, but are licentiates in dentistry only, or by others who hold no registerable medical or dental qualification, but are licensed herbalists, chemists, druggists, bone-setters, patent-medicine vendors, barbers, etc. That those of the first class should treat the constitutional maladies of their patients, one can understand. But for those of the second class to attempt constitutional treatment is most certainly highly improper, and likely to often lead to the most serious consequences. We have, as stated before, no intention of offering a slight to the license in dental surgery, which is an excellent diploma as far as it goes; but we hold that it should be employed strictly for the purpose for which it was intended—viz., as a license for the practise of dentistry. —*London Specialist.*

---

*American Dental Association.*

